# Luteolin Enhances Endothelial Barrier Function and Attenuates Myocardial Ischemia–Reperfusion Injury via FOXP1-NLRP3 Pathway

**DOI:** 10.3390/ijms27020874

**Published:** 2026-01-15

**Authors:** Hanyan Xie, Xinyi Zhong, Nan Li, Mijia Zhou, Miao Zhang, Xiaomin Yang, Hui Wang, Yu Yan, Pengrong Gao, Tianhua Liu, Qiyan Wang, Dongqing Guo

**Affiliations:** 1 School of Life Sciences, Beijing University of Chinese Medicine, Beijing 100029, China; xiehanyanbucm@163.com (H.X.); 20230931146@bucm.edu.cn (X.Z.); 19106413913@163.com (N.L.); 15703905651@163.com (M.Z.); yangxm@bucm.edu.cn (X.Y.); whbucm@163.com (H.W.); 2 College of Chinese Medicine, Beijing University of Chinese Medicine, Beijing 100029, China; 13121153055@163.com (M.Z.); yanyuzi999@outlook.com (Y.Y.); 18611191319@163.com (P.G.); liutianhua@bucm.edu.cn (T.L.)

**Keywords:** myocardial ischemia–reperfusion injury, endothelial barrier function, FOXP1, NLRP3, luteolin

## Abstract

As a natural flavonoid, the flavonoid luteolin is characterized by its powerful antioxidant and anti-inflammatory effects. While its precise mechanisms require further elucidation, existing evidence confirms its efficacy in ameliorating myocardial ischemia–reperfusion injury (MIRI). This research was designed to investigate the mechanism through which luteolin protects against MIRI. We established MIRI rat models through the ligation of left anterior descending coronary artery (LAD). To evaluate the cardioprotective effects of luteolin, echocardiographic analysis was performed, Hematoxylin and Eosin (HE) staining, and serum cardiac injury markers creatine kinase-MB (CK-MB) and lactate dehydrogenase (LDH). Cardiac vascular permeability was determined using Evans blue staining. To mimic ischemia–reperfusion injury, endothelial cells (ECs) were subjected to oxygen-glucose deprivation/reoxygenation (OGD/R) in vitro. Endothelial cell barrier function was evaluated through F-actin phalloidin staining and FITC-Dextran fluorescence leakage experiments. To elucidate the molecular mechanism, FOXP1 small interfering RNA (siRNA) and NLRP3 inhibitor MCC950 were administered. In MIRI rats, luteolin significantly improved cardiac function and preserved endothelial barrier integrity. These effects were associated with upregulation of FOXP1 and suppression of NOD-like receptor family pyrin domain containing 3 (NLRP3) inflammasome. In OGD/R-treated endothelial cells, luteolin restored barrier function and cell viability. The protective effects of luteolin were abolished after FOXP1 silencing. Pharmacological NLRP3 inhibition (MCC950) mirrored luteolin’s protection. Our study indicates that luteolin enhances endothelial barrier function and attenuates MIRI via the FOXP1-NLRP3 pathway. The current study provides a potential drug for MIRI treatment.

## 1. Introduction

Myocardial ischemia–reperfusion injury (MIRI) presents as an important and serious complication for patients undergoing revascularization therapy following myocardial infarction [1]. Despite successful reperfusion, many patients fail to achieve complete cardiac functional recovery, with some developing exacerbated tissue injury [2]. Notably, myocardial ischemia–reperfusion injury (MIRI) is responsible for up to 50% of the ultimate infarct size [3]. Current therapeutic strategies, such as diuretics, oxygen free radical scavengers, beta-blockers and cardioprotective agent, have demonstrated limited efficacy [4,5,6,7]. Thus, research into the pathophysiology of MIRI and develop new drugs for its treatment are imperative.

The pathophysiology of MIRI is multifaceted, driven by a cascade of events that includes calcium overload, heightened oxidative stress, inflammation, and compromised endothelial barrier function [8,9]. Endothelial barrier is a specialized structure formed by endothelial cells between the vascular lumen and vascular wall [10]. It exhibits selective permeability and is crucial for maintaining homeostasis among blood vessels, tissues, and organs [11,12]. Dysfunction of endothelial barrier has been identified as a key pathogenic factor in a number of diseases [13]. During MIRI, impairment of endothelial barrier function leads to increased vascular permeability, leukocyte extravasation, tissue edema and amplified inflammatory responses, which collectively exacerbate myocardial injury [14].

Vascular endothelial cells have a high expression of forkhead box protein P1 (FOXP1), which plays an essential role in governing transcription for heart development. FOXP1 could control cell division and proliferation [15,16,17,18]. However, whether FOXP1 is involved in the endothelial barrier dysfunction in the MIRI remains unclear. Tao Zhuang found that FOXP1 negatively regulated the inflammasome complex, which leads to the suppression of atherosclerotic onset and progression [19]. In the process of sterile inflammation and cardiovascular disease, NOD-like receptor family pyrin domain containing 3 (NLRP3) is the primary mediator [20]. Caspase activation is dependent upon the NLRP3 inflammasome-mediated recruitment of ASC and pro-caspase-1. Proinflammatory cytokines like IL-1β are cleaved and released more readily when caspase-1 is activated [21,22]. In endothelial cells, NLRP3 inflammasome activation thereby drives a series of events, culminating in enhanced inflammatory cascades and cellular damage, resulting in endothelial barrier dysfunction including actin stress fibers forming, alterations involving cytoskeletal reorganization and endothelial cell contraction and increasing endothelial permeability [13,23,24].

It has been demonstrated that traditional Chinese herbal medicine works well for treating heart conditions [25]. A naturally occurring flavonoid compound with anti-inflammatory, anti-cancer, and antioxidant properties, luteolin is present in many plants [26]. Recent studies underscore its direct protective role in endothelium; for instance, luteolin was shown to attenuate enhanced endothelial permeability by inhibiting Protein Kinase C activity [27] and intracellular calcium concentration [28] in human umbilical vein endothelial cells. It has been shown that luteolin exerts protective effects against MIRI [29]. Moreover, emerging research on flavonoid biology reveals their capacity to modulate endothelial inflammasome signaling through transcriptional and post-transcriptional mechanisms. For example, the flavonoid dihydromyricetin suppresses endothelial NLRP3 inflammasome activation by promoting mitophagy, an upstream regulatory pathway [30]. However, it remains unclear whether luteolin enhances endothelial barrier function in MIRI specifically via the transcriptional regulator FOXP1 and its downstream control of the NLRP3 inflammasome. In this paper, this study aimed to elucidate the impact of luteolin on endothelial barrier function by modulating the FOXP1-NLRP3 pathway, utilizing both in vivo MIRI and in vitro OGD/R models.

## 2. Results

### 2.1. Luteolin Improved Cardiac Function in MIRI Rats

**Figure 1:**
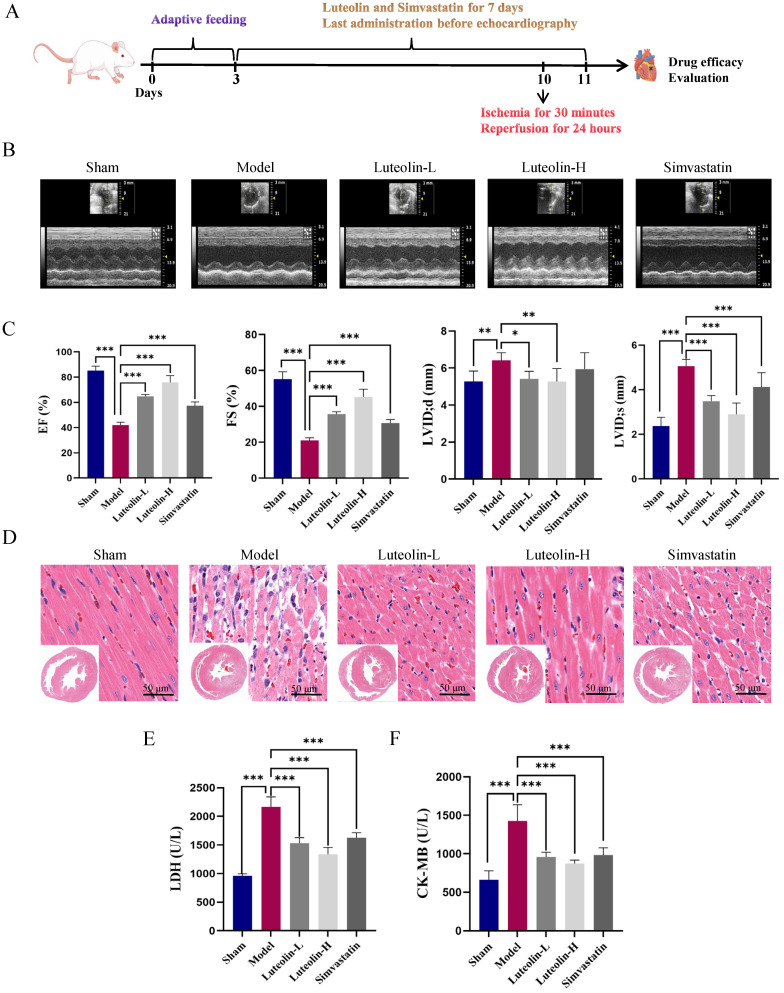
In MIRI rats, luteolin enhanced cardiac function. (**A**) Luteolin treatment experiment using the MIRI rat model. (**B**) Representative echocardiography pictures for every group. (**C**) Quantitative analysis of EF, FS, LVID;d, and LVID;s by echocardiography in each group (*n* = 8 independent rats per group). (**D**) Representative images of HE staining of myocardial tissue, scale bar = 50 μm (*n* = 3 independent rats per group). (**E**,**F**) Levels of LDH and CK-MB in rat serum (*n* = 8 independent rats per group). Data for each group were represented as mean ± SD; * *p* < 0.05, ** *p* < 0.01, *** *p* < 0.001 vs. MIRI model group.

The experimental protocol described was followed to establish rat MIRI model (Figure 1A). To assess the effects of luteolin on MIRI-induced cardiac function, echocardiographic evaluation was performed in rats. The MIRI group showed decreased ejection fraction (EF) and fractional shortening (FS), as well as increased left ventricular end-diastolic diameter (LVID;d) and left ventricular end-systolic diameter in systole (LVID;s). Luteolin administration effectively improved cardiac function. In particular, the high-dose (Figure 1B,C). HE staining showed that the MIRI group’s myocardial tissue architecture was severely disrupted, but this was lessened by luteolin (Figure 1D). Luteolin, at doses of 40 and 80 mg/kg, significantly suppressed the serum release of creatine kinase-MB (CK-MB) and lactate dehydrogenase (LDH) (Figure 1E,F). All of these findings showed that luteolin improved MIRI rats’ cardiac function.

### 2.2. Luteolin Enhanced Cardiac Endothelial Barrier Function in MIRI Rats

**Figure 2:**
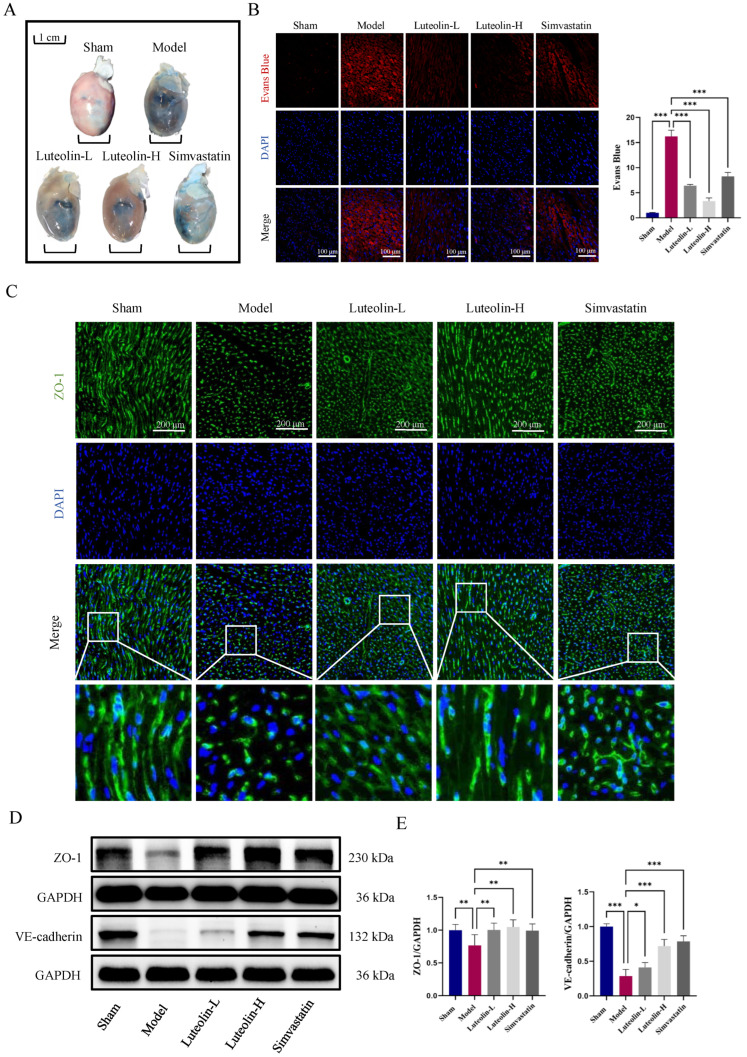
Luteolin improved cardiac endothelial barrier function in MIRI rats. (**A**) Evans blue staining representative of each experimental group (*n* = 3 independent rats per group). (**B**) Fluorescence staining results of cardiac tissue sections after Evans blue perfusion and quantitative analysis, scale bar = 100 μm. (**C**) Immunofluorescence micrographs depicting ZO-1 expression in the different treatment groups, scale bar = 200 μm (*n* = 3 independent rats per group). (**D**,**E**) Representative immunoblots and quantification of ZO-1 and VE-cadherin in each group (*n* = 6 independent rats per group). The data for each group were shown as the mean ± SD; * *p* < 0.05, ** *p* < 0.01, *** *p* < 0.001 vs. MIRI model group.

To assess cardiac vascular leakage in MIRI rats, we employed the Evans blue dye extravasation method. Compared with the sham group, the MIRI group demonstrated substantially elevated vascular permeability, implying a compromise in endothelial barrier integrity. Interestingly, treatment with luteolin (both low and high doses) and Simvastatin markedly reduced vascular leakage (Figure 2A,B). It is known that tight junctions (TJs) and adherens junctions (AJs) are critical for maintaining endothelial integrity. Thus, we examined the distribution of zonula occludens-1 (ZO-1), a key TJ protein, using immunohistochemistry (IF) staining. In the MIRI group, ZO-1 exhibited a discontinuous, irregular distribution. In contrast, luteolin-treated groups showed ZO-1 expression with a more continuous and organized distribution along cell borders (Figure 2C). VE-cadherin is a major AJ protein. As evidenced by Western blot, luteolin treatment effectively recovered the levels of ZO-1 and VE-cadherin, which were diminished in the MIRI group (Figure 2D,E). In conclusion, these findings showed that luteolin improved MIRI rats’ endothelial barrier function.

### 2.3. Luteolin Regulated the FOXP1-NLRP3 Pathway in MIRI Rats

**Figure 3:**
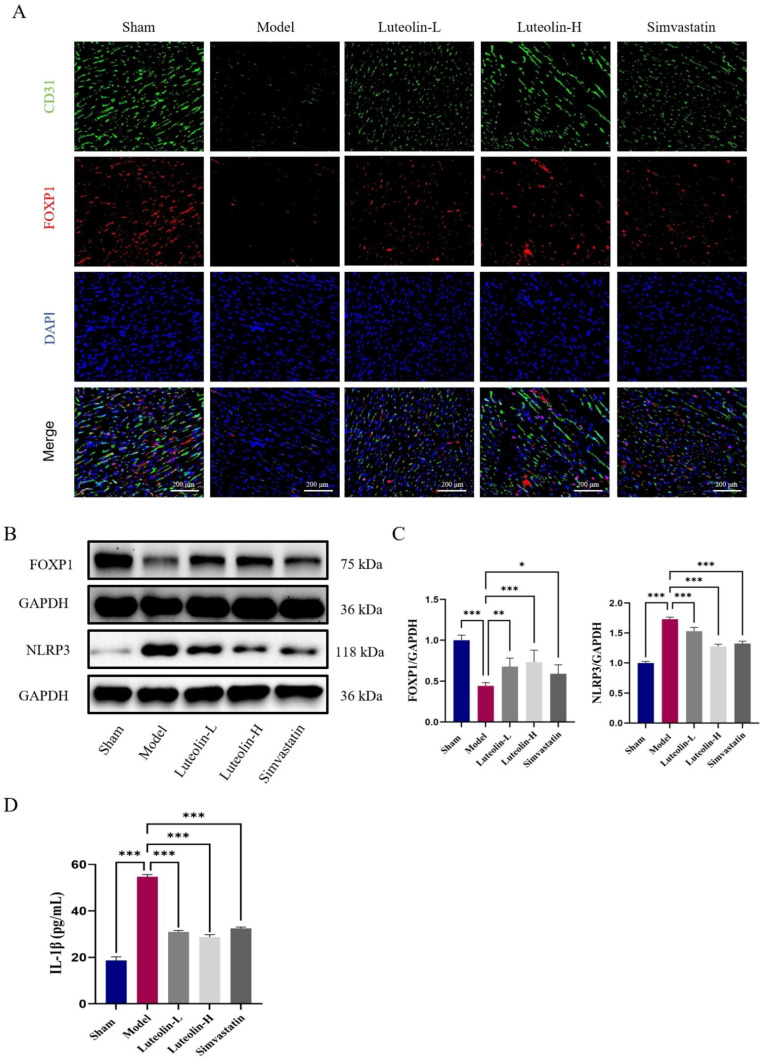
Luteolin regulated the FOXP1-NLRP3 pathway in MIRI rats. (**A**) Representative IF double staining images of FOXP1 and CD31 in each group. Scale bar = 200 μm (*n* = 3 independent rats per group). (**B**,**C**) Representative immunoblotting and quantitative analysis of FOXP1 and NLRP3 in each group (*n* = 6 independent rats per group). (**D**) ELISA detection of the content of inflammatory factor IL-1β in serum (*n* = 6 independent rats per group). The data for each group were shown as the mean ± SD; * *p* < 0.05, ** *p* < 0.01, *** *p* < 0.001 vs. MIRI model group.

IF double-labeling revealed that the endothelial cells in the rat heart sections had high levels of FOXP1expression (Figure 3A). While luteolin administration markedly increased the expression of FOXP1 in endothelial cells, FOXP1 expression was downregulated in the MIRI group (Figure 3A). Western blotting (WB) results showed that luteolin could upregulate FOXP1 expression while suppress NLRP3 expression (Figure 3B,C). Additionally, compared to the MIRI group, luteolin treatment may lower serum levels of the inflammatory cytokine IL-1β (Figure 3D). In summary, luteolin could alleviate MIRI in rats by regulating the FOXP1-NLRP3 pathway in vivo.

### 2.4. Luteolin Ameliorated Cell Viability and Endothelial Barrier Function In Vitro

**Figure 4:**
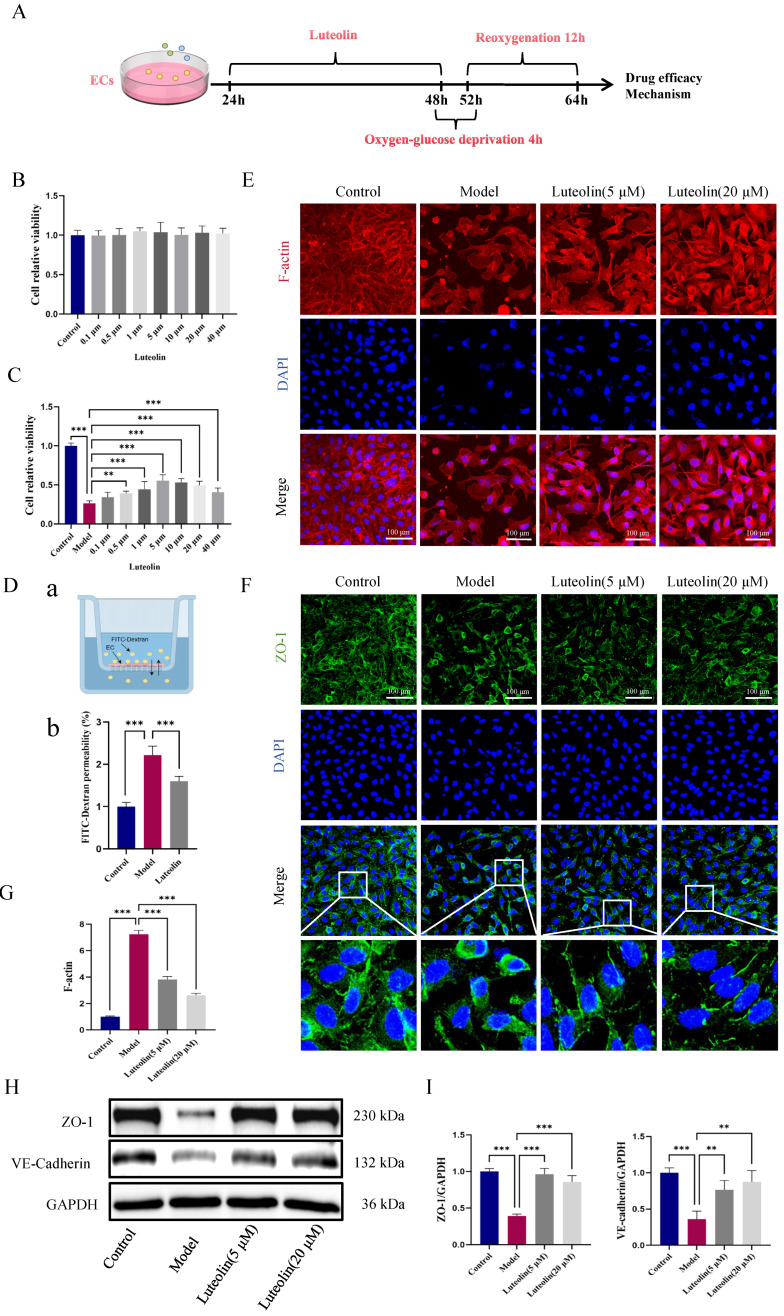
In vitro, luteolin exerted protective effects on both cellular viability and endothelial barrier function. (**A**) A schematic figure of luteolin treatment in an OGD/R-induced endothelial cell injury model. (**B**) Luteolin (0.1 μM, 0.5 μM, 1 μM, 5 μM,10 μM, 20 μM, 40 μM) showed no cytotoxicity to endothelial cells (*n* = 6 independent cell culture experiments). (**C**) Luteolin improved endothelial cell viability in OGD/R treatment (*n* = 6 independent cell culture experiments). (**D**) (**a**) is FITC-Dextran fluorescence penetration model and (**b**) is permeability of each group (*n* = 4 independent cell culture experiments). (**E**,**G**) Representative images of phalloidin staining of endothelial cells treated with luteolin (5 μM and 20 μM) and quantitative analysis. Scale bar = 100 μm (*n* = 3 independent cell culture experiments). Red indicates F-actin (cytoskeleton). (**F**) Representative IF staining images showing ZO-1 (green, tight junctions) and nuclei (blue, DAPI). Scale bar = 100 μm (*n* = 3 independent cell culture experiments). (**H**,**I**) Representative blots of ZO-1 and VE-cadherin in each group and quantitative analysis (*n* = 3 independent cell culture experiments). Data were displayed as the mean ± SD; ** *p* < 0.01, *** *p* < 0.001 vs. OGD/R model group.

We generated an OGD/R-induced endothelial injury model to examine the influence of luteolin (Figure 4A). Cell Counting Kit-8 (CCK-8) assays demonstrated that luteolin (0.1–40 μM) was non-cytotoxic to endothelial cells (Figure 4B). In OGD/R model, luteolin improved cell viability dose-dependently and 5 μM had the optimal efficacy (Figure 4C). The FITC-dextran fluorescence permeability assay was used to assess endothelial barrier function in vitro. A markedly lower FITC-dextran permeability coefficient was observed in luteolin-treated cells relative to the OGD/R group, which implied that luteolin mitigated endothelial barrier dysfunction caused by OGD/R (Figure 4D). F-actin reorganization is frequently linked to impaired endothelial barrier function. Phalloidin staining revealed that luteolin regulated OGD/R-induced F-actin reorganization and restored cell morphology (Figure 4E,G). IF staining of ZO-1 exhibited increased relatively complete ring-like connections, indicating enhanced tight junction integrity (Figure 4F). Luteolin dramatically increased the expression of both ZO-1 and VE-cadherin junctional proteins, according to additional WB analysis (Figure 4H,I). In summary, these findings demonstrated that luteolin could enhance the cell viability and endothelial barrier function in vitro.

### 2.5. FOXP1 Mediated the Protective Effects of Luteolin Against Endothelial Barrier Dysfunction In Vitro

**Figure 5:**
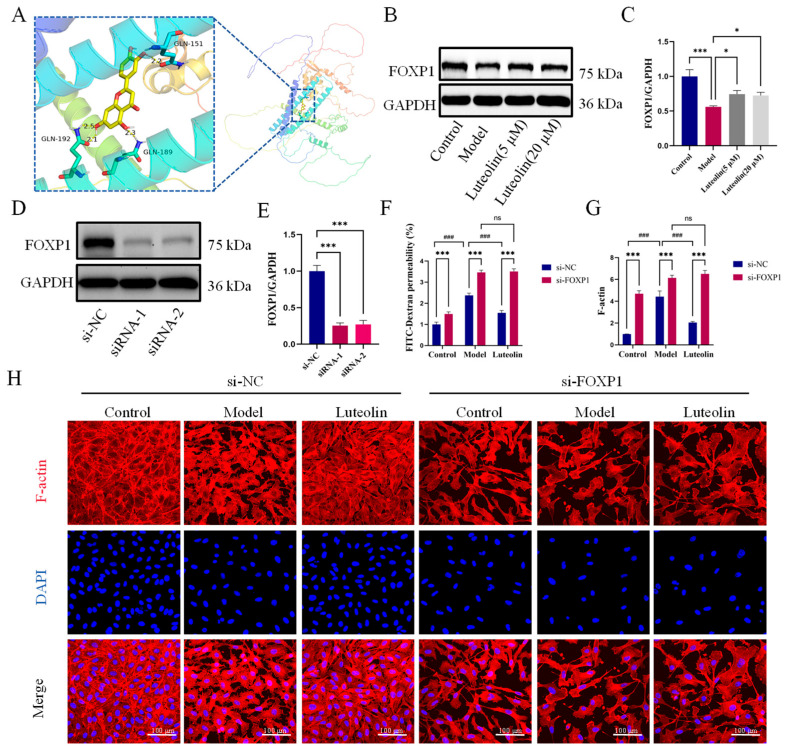

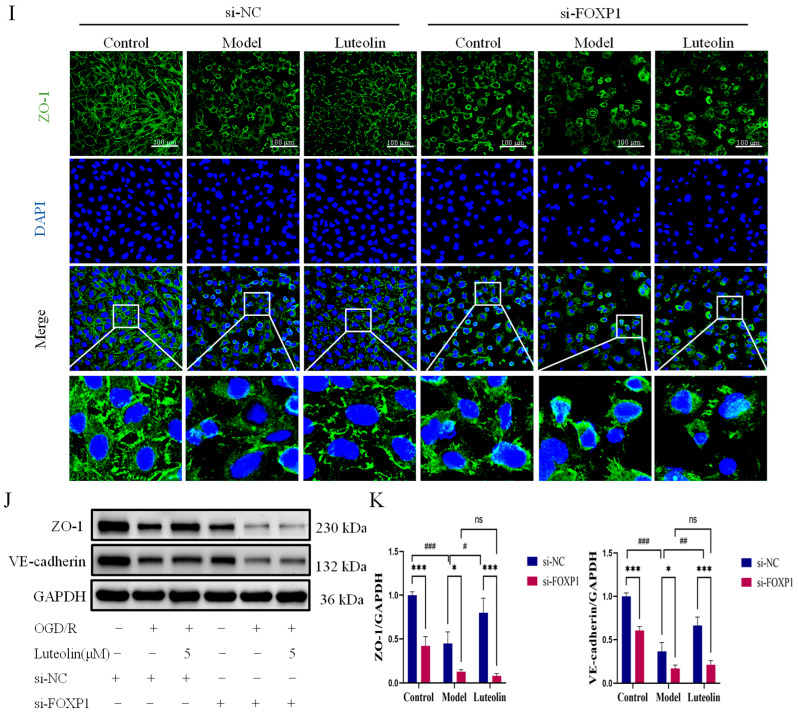
The improvement of endothelial barrier function by luteolin was dependent on FOXP1. (**A**) The docking results and interaction pattern of FOXP1 with luteolin molecules. (**B**,**C**) FOXP1 protein expression detected by immunoblotting, showing representative bands and densitometric quantification for all groups (*n* = 3 independent cell culture experiments). (**D**,**E**) FOXP1 silencing efficacy validated by immunoblotting and quantitative analysis using two distinct siRNAs (*n* = 3 independent cell culture experiments). (**F**) FITC-Dextran fluorescence penetration model and permeability of each group. The concentration of luteolin was 5 μM (*n* = 6 independent cell culture experiments). (**G**,**H**) Representative images of phalloidin staining of endothelial cells treated with luteolin (5 μM, 20 μM) and area of cell F-actin, scale bar = 100 μm (*n* = 3 independent cell culture experiments). (**I**) Representative IF staining images of ZO-1, scale bar = 100 μm (*n* = 3 independent cell culture experiments). (**J**,**K**) Representative immunoblotting and quantitative analysis of ZO-1 and VE-cadherin in each group (*n* = 3 independent cell culture experiments). The data for each group were shown as the mean ± SD; # *p* < 0.05, ## *p* < 0.01, ### *p* < 0.001 vs. OGD/R model group; * *p* < 0.05, *** *p* < 0.001 vs. si-NC group, ns indicates no statistical significance.

We used molecular docking to assess the interaction between luteolin and FOXP1. A strong binding affinity between luteolin and FOXP1 was indicated by the binding energy of −6.4 Kcal/mol. Luteolin could form hydrogen bonds with GLN-192, GLN-189, and GLN-151 of FOXP1, with hydrogen bond lengths of 2.5 Å, 2.1 Å, 2.3 Å, and 2.2 Å (Figure 5A). WB showed that luteolin could promote FOXP1 expression (Figure 5B,C). To validate the role of FOXP1, we used specific siRNAs to knock down its expression. This knockdown was confirmed at the protein level by Western blot analysis (Figure 5D,E). Based on its superior efficacy, siRNA-1 was selected for all subsequent functional experiments. After si-FOXP1, the ability of luteolin to reduce FITC-dextran permeability was abolished (Figure 5F). Furthermore, Phalloidin and ZO-1 staining showed that luteolin failed to reverse the cytoskeletal arrangement and tight junction integrity after si-FOXP1 (Figure 5G–I). Luteolin’s inability to boost ZO-1 and VE-cadherin expression following si-FOXP1 was further verified by WB analysis (Figure 5J,K). These results showed that luteolin’s protective effects against endothelial barrier dysfunction depended on FOXP1.

### 2.6. Luteolin Improved Endothelial Barrier Function by Regulating FOXP1-NLRP3 Pathway In Vitro

**Figure 6:**
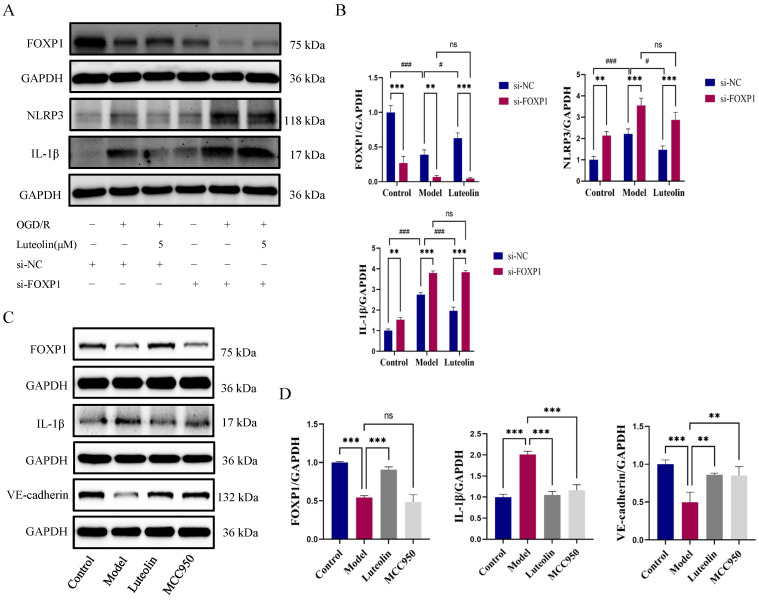
Luteolin improved endothelial barrier function through the FOXP1-NLRP3 pathway in vitro. (**A**,**B**) Western blot analysis of FOXP1, NLRP3, and IL-1β expression in the context of FOXP1 knockdown (*n* = 3 independent cell culture experiments). (**C**,**D**) Representative immunoblotting and quantitative analysis of FOXP1, IL-1β and VE-cadherin in each group with the addition of NLRP3 inhibitor MCC950 (*n* = 3 independent cell culture experiments). The data for each group were shown as mean ± SD; # *p* < 0.05, ** *p* < 0.01, *** *p* < 0.001/### *p* < 0.001 vs. OGD/R model group; ** *p* < 0.01, *** *p* < 0.001 vs. si-NC group, ns indicates no statistical significance.

Protein levels of pathway components were assessed by Western blot in the presence or absence of si-FOXP1 across all experimental groups. NLRP3 and IL-1β expression could be reduced by luteolin, but the effects vanished after si-FOXP1 (Figure 6A,B). The NLRP3 inflammasome activation inhibitor MCC950 was administered, to confirm the connection between FOXP1 and NLRP3. Compared to the OGD/R group, luteolin had the similar effects with MCC950. Furthermore, the expression of FOXP1 had no change (Figure 6C,D), indicating NLRP3 was the downstream of FOXP1. These data collectively confirmed that luteolin could improve endothelial barrier function by regulating FOXP1-NLRP3 pathway.

## 3. Discussion

To assess the effects and mechanism of luteolin in the current study, we built an OGD/R cell model and a MIRI rat model. Both in vivo and in vitro, we discovered that luteolin treatment could improve endothelial barrier function and reduce MIRI. By controlling the FOXP1-NLRP3 pathway, luteolin mechanistically enhanced endothelial barrier function. Collectively, we supposed that luteolin was a potential therapeutic drug against MIRI.

MIRI remains a major clinical challenge despite significant advances in coronary interventional and thrombolytic therapies [31]. While these techniques have improved patient outcomes, reperfusion injury continues to pose a serious risk [32]. Therefore, exploring more effective methods for treating MIRI has become a top priority. Cardiac ischemia–reperfusion can be prevented and treated with the unique advantages of traditional Chinese medicine [33,34,35]. Previous studies have shown that luteolin alleviates MIRI [36,37], but its role in regulating endothelial barrier function during MIRI remains unclear. It is noteworthy that the clinical translation of natural flavonoids like luteolin has historically been hampered by pharmacokinetic challenges, including poor solubility, low oral bioavailability, and rapid metabolism. Recent advances in pharmaceutical formulation, such as the development of nanocarriers, liposomes, and solid dispersions, are actively addressing these limitations by enhancing the stability, targeted delivery, and overall therapeutic efficacy of flavonoids [38]. The study extends these earlier findings by systematically demonstrating that luteolin not only ameliorates cardiac injury markers (CK-MB, LDH) and preserves systolic function (EF, FS), but also specifically mitigates vascular hyperpermeability—a hallmark of endothelial barrier disruption. Notably, the reduction in Evans blue extravasation and the restoration of ZO-1 and VE-cadherin expression provide direct structural and functional evidence for endothelial protection, which has been less emphasized in prior luteolin-MIRI studies. Our study provides the first direct evidence that luteolin preserves endothelial barrier integrity in MIRI, extending beyond its previously documented antioxidant and anti-inflammatory properties. The significant improvement in cardiac function among luteolin-treated MIRI rats corroborates earlier research in this field. Mechanistically, we identified that this protection is closely associated with the upregulation of FOXP1 and the subsequent inhibition of the NLRP3 inflammasome cascade. In this study, we found that luteolin reduced vascular leakage and enhanced endothelial barrier integrity. Similar protective effects were observed in OGD/R induced endothelial cells.

The specific function of FOXP1 in endothelial barrier regulation during acute ischemia–reperfusion was previously undefined. Our findings confirm that FOXP1 is a critical mediator of endothelial resilience in myocardial ischemia–reperfusion injury (MIRI), and its downregulation exacerbates barrier dysfunction. This suggests that FOXP1 could serve as a potential therapeutic target for conditions involving endothelial hyperpermeability.

One possible target for treating cardiovascular diseases was FOXP1 [39]. According to reports, FOXP1 suppressed the TGF-β signaling pathway to prevent cardiac fibrosis and pathological remodeling during heart failure [40]. Additionally, FOXP1 promoted angiogenesis in adult rats following myocardial infarction [41]. These pro-angiogenic and anti-fibrotic roles complement our findings on barrier protection, suggesting FOXP1 is a multifaceted regulator of vascular integrity and repair post-injury. In this investigation, we found that FOXP1 plays a role in luteolin’s ability to prevent endothelial barrier dysfunction. Luteolin could raise FOXP1 expression in endothelial cells in OGD/R-induced ECs and MIRI rats. After si-FOXP1 in endothelial cells, the ability of luteolin to enhance endothelial barrier function was abolished. This loss-of-function experiment crucially establishes a causal link between FOXP1 upregulation and luteolin’s endothelial protective effects, moving beyond correlative observations. Emerging evidence suggests that FOXP1 suppresses NLRP3 inflammasome activation, mitigating brain ischemia–reperfusion injury [42]. Consistent with this, we observed elevated NLRP3 and IL-1β levels following FOXP1 silencing. However, treatment with the NLRP3 inhibitor MCC950 did not alter FOXP1 expression, confirming that NLRP3 acts downstream of FOXP1. This hierarchical relationship is further supported by our molecular docking data, which suggests a direct interaction between luteolin and FOXP1, potentially stabilizing FOXP1 or modulating its activity to initiate the inhibitory cascade on NLRP3. Together, our findings demonstrate that the FOXP1-NLRP3 axis is a critical pathway through which luteolin preserves endothelial barrier function during MIRI. This study provides new insights into MIRI pathophysiology and supports the potential therapeutic application of luteolin.

Regarding the specific site of action within the FOXP1-NLRP3 axis, our data provide a clear directional insight. The finding that luteolin upregulates FOXP1 protein expression positions its primary effect at the transcriptional regulatory level. As a transcription factor, elevated FOXP1 would inherently alter the transcription of its target genes, which is consistent with the observed downstream suppression of NLRP3 and IL-1β. Crucially, the loss of luteolin’s protective effects upon FOXP1 knockdown strongly argues that FOXP1 is a necessary mediator, suggesting luteolin acts upstream of this node. However, the possibility of additional, direct interactions between luteolin and inflammasome proteins cannot be entirely excluded and remains an open question for future investigation. Luteolin’s cardioprotective effects are consistent with its established multi-target nature. Beyond the FOXP1-NLRP3 axis identified here, it acts through complementary pathways, including the activation of the Nrf2 antioxidant response and the inhibition of the NF-κB inflammatory cascade [43,44]. It also modulates processes like autophagy and cell survival via the JNK [45] and Hippo-YAP/PI3K/AKT pathways [46]. This pleiotropic activity, simultaneously targeting oxidative stress, inflammation, and cellular homeostasis, underscores its potential as a comprehensive therapeutic agent whose efficacy in MIRI likely arises from the synergy of these multiple mechanisms.

This study still has a number of shortcomings. First off, without additional validation in primary isolated cardiac microvascular endothelial cells, this study mainly examined the effects of luteolin in EA.hy926 endothelial cells. Second, while we focused on the FOXP1-NLRP3 pathway, we cannot exclude the contribution of the alternative pathways mentioned above to the overall protective effect of luteolin. The interplay between these pathways in the context of MIRI warrants further investigation. While our study demonstrates the suppression of NLRP3 and mature IL-1β, future work incorporating analysis of caspase-1 activation or ASC oligomerization would provide additional mechanistic depth to confirm the specific inhibition of inflammasome assembly and activity. Furthermore, a critical consideration is how the identified FOXP1-NLRP3 axis aligns with or distinguishes itself from the conserved biology of flavonoids. Comprehensive analyses indicate that many flavonoids share common protective modules, such as activation of the Nrf2 antioxidant pathway and inhibition of NF-κB-mediated inflammation [47]. However, the specific upregulation of the transcription factor FOXP1 leading to transcriptional repression of the NLRP3 inflammasome, as a primary mechanism for endothelial barrier protection, appears less commonly reported in generalized flavonoid pathway maps. This suggests that the FOXP1-NLRP3 axis may represent a more distinct or context-specific pathway leveraged by luteolin. Future systematic comparative studies with other flavonoids are necessary to definitively establish whether this mechanism is a unique feature of luteolin or a shared but under-characterized pathway within flavonoid biology. Third, the pharmacokinetic properties, optimal dosing window, and long-term safety profile of luteolin in the setting of ischemia–reperfusion have not been evaluated and are critical for any future clinical translation. Moreover, comparing the efficacy and mechanism of luteolin with other known barrier-protective agents in MIRI (e.g., other flavonoids or statins) would help position its therapeutic potential. Finally, the pharmacokinetic properties of luteolin and its potential for clinical application have not yet been evaluated. In order to strengthen the theoretical basis for the clinical prevention and treatment of MIRI, future studies should investigate luteolin’s mechanisms in greater detail. Future work should involve primary endothelial cells, in-depth pharmacokinetic studies, and models exploring the co-modulation of multiple pathways to fully elucidate luteolin’s therapeutic potential.

## 4. Materials and Methods

### 4.1. Reagents

Luteolin (≥98% pure, CAS No.491-70-3) was acquired from Chengdu Desite Biotechnology Co., Ltd. (491-70-3, Chengdu, China). Simvastatin tablets were purchased from Hangzhou Merck Pharmaceutical Co., Ltd. (Hangzhou, China). MCC950 was purchased from Beijing Biorain Technology Co., Ltd. (AbMole, M6164, Beijing, China). Evans Blue (IE0280) was purchased from Beijing Solarbio Science & Technology Co., Ltd. (Beijing, China). Hematoxylin-Eosin (HE) staining kit (C0105S), FITC-dextran (ST2940), Actin-Tracker Red-Rhodamine (C2207S) and Lipo8000TM transfection reagent (C0533) were purchased from Biyuntian Biotechnology (Shanghai, China). PAGE gel rapid preparation kit was purchased from Shanghai Epizyme Biomedical Technology Co., Ltd. (PG112, Shanghai, China). CCK-8 kit (BN15201) was purchased from Biorigin Beijing Biotechnology Co., Ltd. (Beijing, China). Lactate dehydrogenase LDH (A020-2) and creatine kinase isoenzyme CK-MB (E006-1-1) assay kits were purchased from Nanjing Jiancheng Bioengineering Institute (Nanjing, China). IL-1β ELISA Kit (RA20020) was purchased from Wuhan Bioswamp Biotechnology Co., Ltd. (Wuhan, China).

### 4.2. Animal Management

We purchased specific pathogen-free male Sprague-Dawley rats (200–220 g) from SPF Biotechnology Co., Ltd. (Beijing, China; License: SCXK 2019-0010). The animal study protocol received ethical approval (No. BUCM-2024060703-2163) from the Committee of Beijing University of Chinese Medicine. The rats were maintained under standard conditions in the institutional animal facility of Beijing University of Chinese Medicine under standard conditions, with the temperature maintained at (22 ± 2) °C, relative humidity maintained between 60% and 65%, and a 12 h light/dark cycle. The rats underwent adaptive feeding for 3 days. A total of 40 rats were used, with 8 rats in each of the 5 groups.

### 4.3. Myocardial Ischemia–Reperfusion Animal Model and Drug Administration

A total of 40 rats were used and randomly divided into 5 groups (*n* = 8 per group): Sham, MIRI (Model), MIRI + Luteolin (40 mg/kg), MIRI + Luteolin (80 mg/kg), and MIRI + Simvastatin (1.8 mg/kg, positive control). For seven days, luteolin or simvastatin was taken orally once a day. The sham group was given the same quantity of ddH_2_O. The dosage of luteolin were referenced from the literature [48,49]. The dosage of simvastatin was determined using equivalent conversion between humans and rats and was based on clinical application. On day 7, all groups underwent MIRI induction except the sham group [50,51]. Sodium pentobarbital (50 mg/kg) was used to anesthetize the rats, who were then intubated and given mechanical ventilation. Surgical access to the thoracic cavity was achieved via an incision in the 3rd–4th intercostal space on the left side, the left anterior descending coronary artery (LAD) was ligated with a 5-0 suture. Placed gauze at the site of ligation before performing the ligation operation. Myocardial blanching was a sign that myocardial ischemia had been successfully induced. The suture was released to cause reperfusion injury following 30 min of ischemia. Reperfusion was administered for 24 h. The same surgical procedures were performed on the animals in the sham group, with the exception that the suture passed under LAD without being ligated.

### 4.4. Echocardiographic Assessment

Cardiac function was evaluated using echocardiography (Vevo2100, VisualSonics, Toronto, ON, Canada). Isoflurane was inhaled into the rats to induce anesthesia. Echocardiography detected the parasternal short-axis view of the anesthetized rats’ heart. To assess cardiac function, the following parameters were monitored and measured from the short-axis view: left ventricular ejection fraction (EF), fractional shortening (FS), left ventricular internal diameter at end-diastole (LVID;d), and left ventricular internal diameter at end-systole (LVID;s).

### 4.5. Evans Blue Staining

A 2% Evans blue dye solution (4 mL/kg) was administered to the rats via tail vein injection. After 30 min, the heart was perfused with regular saline until the systemic circulation was free of dye residue from the aortic arch. Hearts were then harvested, and the left ventricle was transversely sectioned. The cross-sectional area of Evans blue dye extravasation in the left ventricle was photographed and quantified using ImageJ software (version 1.54, National Institutes of Health, Bethesda, MD, USA). The region of interest (ROI) was defined as the entire left ventricular area on the cross-section. Vascular leakage was expressed as the percentage of Evans blue-stained area relative to the total left ventricular area. Then, tissue samples were fixed in 4% paraformaldehyde, embedded in paraffin, and sectioned. The resulting slides were subsequently observed under a laser confocal microscope (Olympus, Tokyo, Japan). For fluorescence quantification, three random fields within the ischemic area (from the ligation site toward the apex) per section were selected as ROIs. The mean fluorescence intensity per field was measured using ImageJ, and the average value per animal was calculated for statistical analysis.

### 4.6. Detection of LDH, CK-MB and IL-1β

After echocardiogram, the rats received an intraperitoneal injection of sodium pentobarbital for anesthesia. Vacuum blood collection tubes were then used to draw blood from the abdominal aorta, and the tubes were left at room temperature for an hour. The serum was then extracted from the blood by centrifuging it for ten minutes at 4 °C at 3000 r/min. We measured the serum using commercial kits as per the manufacturer’s guidelines. Lactate dehydrogenase (LDH) activity was measured using a biochemical assay kit (Catalog No. A020-2) from Nanjing Jiancheng Bioengineering Institute. Creatine kinase isoenzyme-MB (CK-MB) activity was measured using a biochemical assay kit (Catalog No. E006-1-1) from Nanjing Jiancheng Bioengineering Institute. Interleukin-1β (IL-1β) levels were quantified using a Rat IL-1 beta ELISA Kit (Catalog No. RA20020) from Abcam (Wuhan, China).

### 4.7. Hematoxylin and Eosin (HE) Staining

Rat hearts were removed, embedded in paraffin, fixed in 4% paraformaldehyde, and cut to a thickness of 5 μm. Hematoxylin dye was applied to the paraffin sections for ten min, followed by two minutes of eosin dye staining. Under a microscope, pictures were taken and observed.

### 4.8. Molecular Docking

Download the SDF format structure of luteolin from the PubChem database (https://pubchem.ncbi.nlm.nih.gov/, accessed on 10 August 2025) and import it into ChemBio3D Ultra 14.0 software for energy minimization optimization. After processing with AutodockTools-1.5.6, the file was saved in “pdbqt” format. The target protein was purified of impurities using PyMOL 2.3.0 software and then imported into AutodockTools-1.5.6 for processing. POCASA 1.1 (http://altair.sci.hokudai.ac.jp/g6/Research/POCASA_e.html, accessed on 10 August 2025) was used to predict protein binding sites, followed by molecular docking with AutoDock Vina 1.1.2. The interaction patterns between the two were analyzed using PyMOL 2.3.0 for the docking results.

### 4.9. Immunofluorescence (IF) Analysis

Sections of paraffin were blocked with sealant and dewaxed. Antibodies ZO-1 (Proteintech, Wuhan, China, 21773-1-AP, 1:200), CD31 (ABclonal, Wuhan, China, A27114, 1:200), and FOXP1 (ABclonal, A23442, 1:50) were incubated overnight at 4 °C in a wet box. After three PBS washes, the slides were incubated with the secondary antibody (Abways, Shanghai, China, AB0152, AB0141, 1:500). The cell nuclei were labeled with DAPI.

Following seeding on confocal culture dishes, cells were blocked with a blocking buffer containing 5% goat serum, fixed with 4% paraformaldehyde. Antibody ZO-1 (Proteintech, 21773-1-AP, 1:200) was used to incubate cells for an entire night at 4 °C. The cells were then exposed to the secondary antibody for an hour at room temperature in the dark. The cell nuclei were labeled with DAPI.

### 4.10. Western Blot Analysis

We lysed myocardial tissue and endothelial cells with RIPA lysis buffer containing both protease and phosphatase inhibitors. The supernatant was then collected by centrifugation. Western blot was used to measure the expression of the protein. The following antibodies were used: ZO-1 (Proteintech, 21773-1-AP, 1:5000), VE-cadherin (Santa Cruz, sc-9989, 1:500), FOXP1 (ABclonal, A23442, 1:3000), NLRP3 (Abcam, ab263899, 1:1000), IL-1β (Abcam, ab283818, 1:1000), GAPDH (Proteintech, 60004-1-Ig, 1:50,000), HRP-conjugated goat anti-mouse (Abways, AB0102, 1:10,000) and HRP-conjugated goat anti-rabbit (Abways, AB0101, 1:10,000) secondary antibodies. Target bands intensities were analyzed and quantified using the ImageJ software.

### 4.11. Cell Culture

Under standard cell culture conditions (37 °C, 5% CO_2_), EA.hy926 endothelial cells (Dalian Meilun Biotechnology Co., Ltd., Dalian, China) were routinely cultured in DMEM containing 10% fetal bovine serum and 1% penicillin/streptomycin.

### 4.12. The OGD/R Induced Endothelial Injury Model

To cause endothelial damage in vitro, the OGD/R model was developed [52,53]. Endothelial cells were seeded into culture dishes at a density of 8 × 10^4^/mL. Following a 48 h period, the cells were incubated for four hours in an anaerobic chamber using Earle’s balanced salt solution (EBSS). The cells were replaced for 12 h to reoxygenate. 5 μM and 20 μM luteolin were added to the luteolin group for 24 h before OGD/R.

### 4.13. Cell Viability Assay

The manufacturer’s instructions were followed when seeding cells into 96-well plates. Using a microplate reader (SpectraMax i3x, Molecular Devices, San Jose, CA, USA), the absorbance at 450 nm was recorded subsequent to a 2 h incubation period at 37 °C.

### 4.14. Phalloidin Staining

The confocal dishes were seeded with endothelial cells. After two PBS washes, the cells were fixed for 15 min using an immunostaining fixation solution. Following three rounds of immunostaining wash solution, the cells were incubated with rhodamine phalloidin (1:100). Cell nuclei were labeled with DAPI following three rounds of washing with the PBS wash solution. The samples were examined, using a confocal laser scanning microscope (Olympus, Japan), we captured the fluorescent signals.

### 4.15. FITC-Dextran Fluorescence Permeability Assay

The transwell’s upper chamber (JET BIOFIL, Guangzhou, China, TCS016024) was filled with endothelial cells, while we added complete medium to the lower compartment. After 48 h at 37 °C, the cells reached 100% confluence. Various treatments were applied to the cells. In order to measure permeability, the upper chamber was filled with FITC-dextran (1 mg/mL in serum-free DMEM), while the lower compartment loaded with serum-free DMEM. Following a 45 min incubation at 37 °C, 100 μL aliquots from both chambers were transferred to a black 96-well plate. Fluorescence intensity was recorded on a SpectraMax i3x microplate reader (Molecular Devices, USA) with wavelengths set at 492 nm (excitation) and 520 nm (emission). For each experimental condition, measurements were performed in 3 technical replicates per independent experiment, and the experiment was repeated independently 4 times (biological replicates).

### 4.16. siRNA Transfection

To further evaluate the role of transcription factor FOXP1, two synthetic siRNAs (GenePharma, Shanghai, China) were used to knock down the expression of FOXP1. The siRNA-1 (5′−3′) sequence was GCAGCAAGUUAGUGGAUUATT; UAAUCCACUAACUUGCUGCTT. The siRNA-2 (5′−3′) sequence was GCUCAUACUGCAGAAGAAATT; UUUCUUCUGCAGUAUGAGCTT. A commercially validated non-targeting scrambled siRNA (si-NC) was used as a negative control to account for any potential off-target effects associated with the transfection procedure and the presence of siRNA duplexes. The sequence of si-NC (5′−3′) was UUCUCCGAACGUGUCACGUTT; ACGUGACACGUUCGGAGAATT. Twenty-four h before transfection, cells were seeded in culture dishes and reached 60–70% confluency. During transfection, the medium was replaced with a transfection mixture contain Lipo8000TM, siRNA, and serum-free DMEM. The transfection mixture was taken out and replaced with new medium containing 10% FBS after the cells were incubated at 37 °C for six h. Following transfection, the endothelial cells were cultured for 24 h at 37 °C. Western blot was used to confirm the knockdown effect before moving on to other experiments.

### 4.17. Statistical Analysis

All data are presented as the mean ± standard deviation (SD). The normality of data distribution was assessed using the Shapiro–Wilk test. For comparisons between two groups, an unpaired two-tailed Student’s *t*-test was applied. For comparisons among three or more groups, one-way or two-way analysis of variance (ANOVA) was used, as appropriate for the experimental design. When ANOVA indicated significant differences, Tukey’s post hoc test was employed for multiple comparisons between individual groups, with correction for the family-wise error rate. A *p*-value of less than 0.05 (*p* < 0.05) was considered statistically significant. The sample size ‘n’ reported in the figure legends represents the number of biological replicates (i.e., independent animals or independently performed cell culture experiments). Within each biological replicate, technical replicates (e.g., multiple wells in a plate) were used, and their mean value was taken as a single data point for statistical analysis. All statistical analyses and graph generation were performed using GraphPad Prism software (version 9.5.0).

## 5. Conclusions

This study demonstrates that luteolin effectively attenuates myocardial ischemia–reperfusion injury (MIRI) in both rat and cellular models. We found that luteolin preserves cardiac function, reduces myocardial injury, and most notably enhances endothelial barrier integrity. Mechanistically, these protective effects are mediated through the upregulation of FOXP1 and subsequent inhibition of the NLRP3 inflammasome pathway. Our findings not only elucidate a novel mechanism by which luteolin protects against MIRI but also highlight the FOXP1–NLRP3 axis as a promising therapeutic target for endothelial stabilization during reperfusion injury. These results support further preclinical development of luteolin and suggest that targeting endothelial barrier function may represent a viable strategy for improving outcomes in patients undergoing revascularization therapy.

## Data Availability

The original data presented in the study have been presented in the article, and further data can be requested by directly contacting the corresponding authors.
